# 1,1′-Bis(diisobutyl­phosphino)cobalto­cenium hexa­fluorido­phosphate

**DOI:** 10.1107/S1600536808017194

**Published:** 2008-06-19

**Authors:** Jian-Guo Hou, Ding-Biao Li, Man-Zhen Qiu

**Affiliations:** a Department of Biotic Environment, Nanchang Institute of Technology, Nanchang 330013, People’s Republic of China

## Abstract

In the title compound, [Co(C_13_H_22_P)_2_]PF_6_, the Co^III^ atom is sandwiched between two (diisobutyl­phosphino)cyclo­penta­dienenyl ligands. The two diisobutyl­phophine units are *trans* to each other with respect to the Co^III^ metal center. The PF_6_^−^ anion links the cobaltocenium cations *via* weak C—H⋯F hydrogen bonds into a chain running along the *b* axis. The chains are further linked by C—H⋯F hydrogen bonds, forming a layer extending parallel to the (10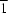) plane.

## Related literature

For background to cobaltocene derivatives applied as catal­ysts, see: Mathews *et al.* (2000 ▶). For the structures of closely related compounds, see: Brasse *et al.* (2000 ▶); Hou *et al.* (2007 ▶).
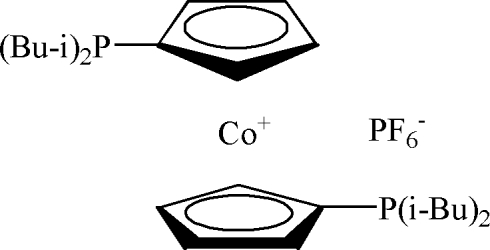


## Experimental

### 

#### Crystal data

[Co(C_13_H_22_P)_2_]·PF_6_
*M* *_r_* = 622.45
Monoclinic, 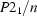

*a* = 16.7733 (3) Å
*b* = 10.4660 (2) Å
*c* = 18.5105 (4) Å
β = 108.288 (1)°
*V* = 3085.38 (10) Å^3^
*Z* = 4
Mo *K*α radiation
μ = 0.76 mm^−1^
*T* = 298 (2) K
0.40 × 0.04 × 0.02 mm

#### Data collection

Bruker SMART CCD area-detector diffractometer
Absorption correction: multi-scan (**SADABS**; Sheldrick, 2004 ▶) *T*_min_ = 0.750, *T*_max_ = 0.985
33818 measured reflections
6733 independent reflections
3878 reflections with *I* > 2σ(*I*)
*R*_int_ = 0.083

#### Refinement

*R*[*F* ^2^ > 2σ(*F* ^2^)] = 0.056
*wR*(*F* ^2^) = 0.147
*S* = 0.97
6733 reflections
333 parameters
H-atom parameters constrained
Δρ_max_ = 0.47 e Å^−3^
Δρ_min_ = −0.34 e Å^−3^

### 

Data collection: *SMART* (Bruker, 2001 ▶); cell refinement: *SAINT* (Bruker, 2001 ▶); data reduction: *SAINT*; program(s) used to solve structure: *SHELXS97* (Sheldrick, 2008 ▶); program(s) used to refine structure: *SHELXS97* (Sheldrick, 2008 ▶); molecular graphics: *SHELXTL* (Sheldrick, 2008 ▶); software used to prepare material for publication: *SHELXTL*.

## Supplementary Material

Crystal structure: contains datablocks I, global. DOI: 10.1107/S1600536808017194/is2295sup1.cif

Structure factors: contains datablocks I. DOI: 10.1107/S1600536808017194/is2295Isup2.hkl

Additional supplementary materials:  crystallographic information; 3D view; checkCIF report

## Figures and Tables

**Table 1:** Hydrogen-bond geometry (Å, °)

*D*—H⋯*A*	*D*—H	H⋯*A*	*D*⋯*A*	*D*—H⋯*A*
C3—H3⋯F5^i^	0.98	2.44	3.237 (5)	138
C15—H15⋯F4^i^	0.98	2.39	3.278 (4)	150
C17—H17⋯F2	0.98	2.51	3.188 (5)	126
C18—H18⋯F6	0.98	2.41	3.305 (4)	152
C19—H19*B*⋯F2^ii^	0.97	2.54	3.494 (4)	167

Symmetry codes: (i) 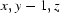; (ii) 
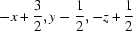
.

**Fig. 1.**
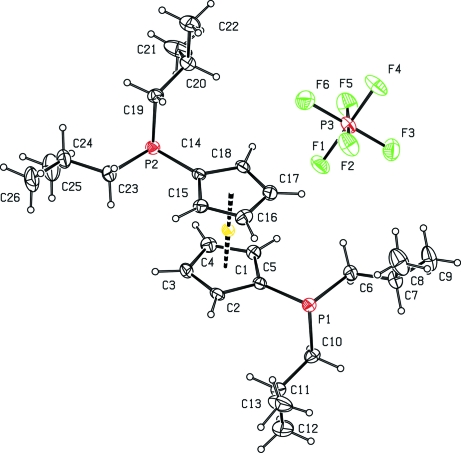
The molecular structure of (I), showing ellipsoids at 30% probability level.

**Fig. 2.**
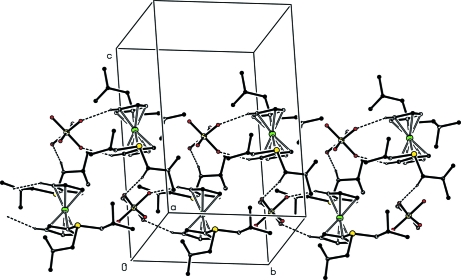
The packing diagram of two-dimensional sheet with hydrogen bonds shown as dashed lines. H atoms not involved in the hydrogen bonds have been omitted for clarity.

## supplementary crystallographic information

### Comment

Cobaltocene derivatives have been applied as catalysts in cross-coupling reactions (Mathews *et al.*, 2000). As part of our investigations of new catalysts, we have focused our attention on cobaltocenium compounds. Some complexes, such as 1,1'-bis(diphenylphosphino)cobaltocenium tetrafluoridoborate, have been obtained and reported (Hou *et al.*, 2007). Herein, we report the structure of the title compound, (I) (Fig. 1). The molecular structure of the title complex consists of the [(η_5_-(*i*-C_4_H_9_)_2_PC_5_H_4_)_2_Co]^+^ cation and the PF_6_^-^ anion, which is very similar to the compounds 1,1'-bis(diphenylphosphino)cobaltocenium tetrafluoridoborate (Hou *et al.*, 2007) and 1,1'-bis(diphenylphosphino)cobaltocenium hexafluorophosphate with different substituents (Brasse *et al.*, 2000). The two (*i*-C_4_H_9_)_2_P substituents are *trans* to each other with respect to the Co^III^ metal center, and the two substituted Cp rings staggered and are essentially parallel with a dihedral angle of 1.8 (2)°. The Co1···*Cg*1 and Co1···*Cg*2 distances are 1.6429 (15) and 1.6430 (3) Å, respectively, and the *Cg*1···Co1···*Cg*2 angle is 179.13 (8)° (*Cg*1 and *Cg*2 are the centroids of the two cyclopentadienyl) The hydrogen bonds of C—H···F play a key role of the stabilization of crystal structure of the title compound. As shown in Fig. 2, there are extensive nonclassical hydrogen bonds formed by C—H···F (Table 2). There are not only intramolecular but also intermolecular hydrogen bonds between the cation and anion, thus, two-dimensional layers extending parallel to the (101) plane were formed.

### Experimental

The title compound was obtained by anion exchange of 1,1'-bis(di-isobutylphosphino)cobaltocenium chloride with ammonium hexaflurophosphate. Crystals appropriate for data collection were obtained by slow diffusion of hexane into a solution of the title compound in dichloromethane at 293 K.

### Refinement

All H atoms were placed in geometrically idealized positions and constrained to ride on their parent atoms with C—H = 0.96–0.98 Å, and with *U*_iso_(H) = 1.2*U*_eq_(C) or 1.5*U*_eq_(methyl C).

### Crystal data

**Table:**

[Co(C_13_H_22_P)_2_]·PF_6_	*F*_000_ = 1304
*M**_r_* = 622.45	*D*_x_ = 1.340 Mg m^−^^3^
Monoclinic, *P*2_1_/*n*	Mo *K*α radiation λ = 0.71073 Å
Hall symbol: -P 2yn	Cell parameters from 3150 reflections
*a* = 16.7733 (3) Å	θ = 2.3–19.9º
*b* = 10.4660 (2) Å	µ = 0.76 mm^−^^1^
*c* = 18.5105 (4) Å	*T* = 298 (2) K
β = 108.288 (1)º	Needle, yellow
*V* = 3085.38 (10) Å^3^	0.40 × 0.04 × 0.02 mm
*Z* = 4	

### Data collection

**Table:**

Bruker SMART CCD area-detector diffractometer	6733 independent reflections
Radiation source: fine-focus sealed tube	3878 reflections with *I* > 2σ(*I*)
Monochromator: graphite	*R*_int_ = 0.083
*T* = 298(2) K	θ_max_ = 27.0º
φ and ω scans	θ_min_ = 1.4º
Absorption correction: multi-scan(*SADABS*; Sheldrick, 2004)	*h* = −21→21
*T*_min_ = 0.750, *T*_max_ = 0.985	*k* = −13→13
33818 measured reflections	*l* = −23→22

### Refinement

**Table:**

Refinement on *F*^2^	Secondary atom site location: difference Fourier map
Least-squares matrix: full	Hydrogen site location: inferred from neighbouring sites
*R*[*F*^2^ > 2σ(*F*^2^)] = 0.056	H-atom parameters constrained
*wR*(*F*^2^) = 0.147	*w* = 1/[σ^2^(*F*_o_^2^) + (0.0731*P*)^2^] where *P* = (*F*_o_^2^ + 2*F*_c_^2^)/3
*S* = 0.97	(Δ/σ)_max_ = 0.001
6733 reflections	Δρ_max_ = 0.47 e Å^−^^3^
333 parameters	Δρ_min_ = −0.34 e Å^−^^3^
Primary atom site location: structure-invariant direct methods	Extinction correction: none

### Special details

**Table:**

Geometry. All e.s.d.'s (except the e.s.d. in the dihedral angle between two l.s. planes) are estimated using the full covariance matrix. The cell e.s.d.'s are taken into account individually in the estimation of e.s.d.'s in distances, angles and torsion angles; correlations between e.s.d.'s in cell parameters are only used when they are defined by crystal symmetry. An approximate (isotropic) treatment of cell e.s.d.'s is used for estimating e.s.d.'s involving l.s. planes.
Refinement. Refinement of *F*^2^ against ALL reflections. The weighted *R*-factor *wR* and goodness of fit *S* are based on *F*^2^, conventional *R*-factors *R* are based on *F*, with *F* set to zero for negative *F*^2^. The threshold expression of *F*^2^ > σ(*F*^2^) is used only for calculating *R*-factors(gt) *etc*. and is not relevant to the choice of reflections for refinement. *R*-factors based on *F*^2^ are statistically about twice as large as those based on *F*, and *R*- factors based on ALL data will be even larger.

### Fractional atomic coordinates and isotropic or equivalent isotropic displacement parameters (Å^2^)

**Table:**

	*x*	*y*	*z*	*U*_iso_*/*U*_eq_	
Co1	0.66580 (3)	0.34637 (4)	0.07609 (2)	0.04054 (16)	
C1	0.6975 (2)	0.3487 (3)	−0.02171 (18)	0.0413 (8)	
C2	0.6752 (2)	0.2221 (3)	−0.00492 (18)	0.0480 (9)	
H2	0.7119	0.1470	0.0038	0.058*	
C3	0.5916 (2)	0.2239 (4)	−0.0027 (2)	0.0560 (10)	
H3	0.5608	0.1507	0.0081	0.067*	
C4	0.5606 (2)	0.3499 (4)	−0.01797 (19)	0.0542 (10)	
H4	0.5047	0.3794	−0.0193	0.065*	
C5	0.6254 (2)	0.4270 (3)	−0.02941 (18)	0.0468 (9)	
H5	0.6215	0.5189	−0.0402	0.056*	
C6	0.7928 (2)	0.5658 (3)	−0.0360 (2)	0.0558 (10)	
H6A	0.7526	0.5870	−0.0849	0.067*	
H6B	0.7717	0.5993	0.0033	0.067*	
C7	0.8757 (3)	0.6294 (4)	−0.0295 (3)	0.0740 (13)	
H7	0.8998	0.5880	−0.0655	0.089*	
C8	0.9371 (3)	0.6159 (6)	0.0495 (3)	0.126 (2)	
H8A	0.9162	0.6608	0.0850	0.189*	
H8B	0.9904	0.6511	0.0507	0.189*	
H8C	0.9439	0.5271	0.0630	0.189*	
C9	0.8631 (3)	0.7712 (4)	−0.0501 (3)	0.1105 (19)	
H9A	0.8379	0.8127	−0.0164	0.166*	
H9B	0.8270	0.7798	−0.1016	0.166*	
H9C	0.9164	0.8099	−0.0451	0.166*	
C10	0.7851 (2)	0.3465 (4)	−0.1272 (2)	0.0562 (10)	
H10A	0.7344	0.3882	−0.1586	0.067*	
H10B	0.8316	0.3803	−0.1419	0.067*	
C11	0.7776 (3)	0.2041 (4)	−0.1449 (2)	0.0694 (12)	
H11	0.7300	0.1710	−0.1306	0.083*	
C12	0.7589 (3)	0.1826 (5)	−0.2301 (3)	0.1059 (18)	
H12A	0.8042	0.2160	−0.2458	0.159*	
H12B	0.7077	0.2256	−0.2573	0.159*	
H12C	0.7530	0.0928	−0.2409	0.159*	
C13	0.8552 (4)	0.1312 (4)	−0.1000 (3)	0.1079 (19)	
H13A	0.8506	0.0440	−0.1170	0.162*	
H13B	0.8603	0.1337	−0.0469	0.162*	
H13C	0.9039	0.1695	−0.1077	0.162*	
C14	0.6338 (2)	0.3542 (3)	0.17449 (18)	0.0406 (8)	
C15	0.7006 (2)	0.2654 (3)	0.18142 (18)	0.0470 (9)	
H15	0.6989	0.1739	0.1921	0.056*	
C16	0.7687 (2)	0.3305 (4)	0.16930 (19)	0.0576 (10)	
H16	0.8224	0.2923	0.1706	0.069*	
C17	0.7466 (2)	0.4595 (4)	0.15524 (19)	0.0574 (10)	
H17	0.7821	0.5269	0.1450	0.069*	
C18	0.6640 (2)	0.4751 (3)	0.15777 (18)	0.0493 (9)	
H18	0.6323	0.5554	0.1492	0.059*	
C19	0.5497 (2)	0.3896 (3)	0.28119 (19)	0.0528 (9)	
H19A	0.5086	0.3520	0.3018	0.063*	
H19B	0.6046	0.3598	0.3120	0.063*	
C20	0.5469 (3)	0.5333 (4)	0.2898 (2)	0.0699 (12)	
H20	0.5877	0.5708	0.2680	0.084*	
C21	0.4615 (4)	0.5887 (5)	0.2477 (3)	0.114 (2)	
H21A	0.4208	0.5578	0.2702	0.171*	
H21B	0.4455	0.5632	0.1953	0.171*	
H21C	0.4640	0.6803	0.2510	0.171*	
C22	0.5730 (3)	0.5705 (5)	0.3741 (2)	0.0917 (15)	
H22A	0.5717	0.6618	0.3786	0.138*	
H22B	0.6288	0.5401	0.3993	0.138*	
H22C	0.5348	0.5329	0.3973	0.138*	
C23	0.5366 (2)	0.1557 (3)	0.2033 (2)	0.0605 (10)	
H23A	0.5477	0.1120	0.1612	0.073*	
H23B	0.5842	0.1405	0.2484	0.073*	
C24	0.4590 (3)	0.0970 (4)	0.2154 (3)	0.0708 (12)	
H24	0.4482	0.1431	0.2575	0.085*	
C25	0.3822 (3)	0.1080 (5)	0.1483 (3)	0.115 (2)	
H25A	0.3907	0.0632	0.1060	0.172*	
H25B	0.3711	0.1964	0.1352	0.172*	
H25C	0.3354	0.0714	0.1602	0.172*	
C26	0.4769 (3)	−0.0421 (4)	0.2402 (4)	0.113 (2)	
H26A	0.4304	−0.0761	0.2538	0.169*	
H26B	0.5269	−0.0464	0.2832	0.169*	
H26C	0.4846	−0.0910	0.1990	0.169*	
F1	0.63466 (15)	0.7385 (2)	0.03501 (14)	0.0871 (8)	
F2	0.75008 (15)	0.7617 (2)	0.13489 (16)	0.0953 (9)	
F3	0.71730 (17)	0.9112 (3)	0.04222 (16)	0.0973 (8)	
F4	0.70793 (18)	0.9559 (2)	0.15705 (15)	0.1059 (10)	
F5	0.59126 (16)	0.9334 (2)	0.05722 (17)	0.1051 (9)	
F6	0.62383 (18)	0.7849 (3)	0.14848 (15)	0.0981 (8)	
P1	0.80124 (6)	0.39039 (9)	−0.02673 (5)	0.0469 (3)	
P2	0.52962 (6)	0.32919 (9)	0.18315 (5)	0.0476 (3)	
P3	0.67083 (6)	0.84835 (9)	0.09545 (6)	0.0561 (3)	

### Atomic displacement parameters (Å^2^)

**Table:**

	*U*^11^	*U*^22^	*U*^33^	*U*^12^	*U*^13^	*U*^23^
Co1	0.0484 (3)	0.0352 (3)	0.0388 (3)	−0.0031 (2)	0.0149 (2)	−0.0013 (2)
C1	0.0513 (19)	0.0361 (19)	0.0372 (17)	−0.0008 (16)	0.0150 (15)	−0.0037 (15)
C2	0.069 (2)	0.033 (2)	0.044 (2)	−0.0023 (17)	0.0203 (17)	−0.0092 (16)
C3	0.066 (2)	0.052 (3)	0.052 (2)	−0.0226 (19)	0.0213 (19)	−0.0142 (19)
C4	0.048 (2)	0.063 (3)	0.045 (2)	−0.0019 (19)	0.0064 (16)	−0.0064 (19)
C5	0.053 (2)	0.044 (2)	0.0401 (19)	0.0021 (17)	0.0109 (16)	0.0053 (16)
C6	0.061 (2)	0.043 (2)	0.067 (2)	−0.0035 (18)	0.0257 (19)	0.0008 (18)
C7	0.078 (3)	0.057 (3)	0.094 (3)	−0.019 (2)	0.036 (3)	−0.011 (2)
C8	0.089 (4)	0.107 (4)	0.150 (5)	−0.031 (3)	−0.008 (4)	−0.014 (4)
C9	0.123 (4)	0.060 (3)	0.148 (5)	−0.031 (3)	0.041 (4)	0.010 (3)
C10	0.066 (2)	0.062 (3)	0.047 (2)	−0.0002 (19)	0.0268 (18)	0.0003 (19)
C11	0.083 (3)	0.068 (3)	0.072 (3)	−0.021 (2)	0.046 (2)	−0.022 (2)
C12	0.129 (4)	0.121 (5)	0.081 (4)	−0.029 (4)	0.051 (3)	−0.042 (3)
C13	0.163 (5)	0.062 (3)	0.101 (4)	0.027 (3)	0.046 (4)	−0.014 (3)
C14	0.055 (2)	0.0317 (19)	0.0366 (18)	0.0033 (15)	0.0173 (15)	−0.0004 (15)
C15	0.060 (2)	0.043 (2)	0.0408 (19)	0.0072 (17)	0.0193 (17)	0.0054 (16)
C16	0.051 (2)	0.076 (3)	0.043 (2)	0.005 (2)	0.0113 (17)	0.001 (2)
C17	0.067 (3)	0.065 (3)	0.041 (2)	−0.022 (2)	0.0178 (18)	−0.0124 (19)
C18	0.074 (3)	0.034 (2)	0.041 (2)	−0.0064 (17)	0.0211 (18)	−0.0087 (16)
C19	0.063 (2)	0.052 (2)	0.047 (2)	0.0069 (18)	0.0228 (18)	0.0029 (17)
C20	0.094 (3)	0.054 (3)	0.072 (3)	−0.013 (2)	0.041 (2)	−0.013 (2)
C21	0.167 (5)	0.066 (3)	0.091 (4)	0.056 (4)	0.015 (4)	−0.008 (3)
C22	0.105 (4)	0.093 (4)	0.078 (3)	−0.014 (3)	0.030 (3)	−0.035 (3)
C23	0.075 (3)	0.040 (2)	0.074 (3)	−0.0032 (19)	0.034 (2)	0.004 (2)
C24	0.077 (3)	0.052 (3)	0.087 (3)	−0.014 (2)	0.030 (2)	0.004 (2)
C25	0.087 (4)	0.119 (5)	0.116 (4)	−0.041 (3)	0.001 (3)	0.011 (4)
C26	0.109 (4)	0.053 (3)	0.186 (6)	−0.014 (3)	0.061 (4)	0.024 (3)
F1	0.0994 (18)	0.0704 (17)	0.0828 (17)	−0.0229 (14)	0.0160 (14)	−0.0294 (14)
F2	0.0789 (16)	0.0622 (16)	0.120 (2)	0.0124 (13)	−0.0038 (15)	0.0095 (15)
F3	0.105 (2)	0.089 (2)	0.103 (2)	−0.0209 (16)	0.0388 (16)	0.0105 (16)
F4	0.142 (3)	0.0577 (16)	0.096 (2)	−0.0114 (16)	0.0059 (17)	−0.0290 (15)
F5	0.0843 (18)	0.0725 (18)	0.139 (2)	0.0210 (14)	0.0074 (17)	0.0211 (17)
F6	0.128 (2)	0.0788 (19)	0.105 (2)	0.0015 (17)	0.0616 (18)	0.0080 (16)
P1	0.0508 (5)	0.0438 (6)	0.0477 (5)	−0.0007 (4)	0.0179 (4)	−0.0006 (4)
P2	0.0546 (5)	0.0429 (6)	0.0472 (5)	0.0024 (4)	0.0188 (4)	0.0008 (4)
P3	0.0628 (6)	0.0361 (6)	0.0614 (6)	−0.0009 (5)	0.0081 (5)	−0.0028 (5)

### Geometric parameters (Å, °)

**Table:**

Co1—C16	2.028 (3)	C13—H13B	0.9600
Co1—C2	2.028 (3)	C13—H13C	0.9600
Co1—C18	2.033 (3)	C14—C15	1.430 (4)
Co1—C17	2.034 (3)	C14—C18	1.433 (4)
Co1—C15	2.036 (3)	C14—P2	1.823 (3)
Co1—C5	2.038 (3)	C15—C16	1.407 (5)
Co1—C1	2.041 (3)	C15—H15	0.9800
Co1—C3	2.045 (3)	C16—C17	1.403 (5)
Co1—C4	2.053 (3)	C16—H16	0.9800
Co1—C14	2.056 (3)	C17—C18	1.410 (5)
C1—C5	1.431 (5)	C17—H17	0.9800
C1—C2	1.437 (4)	C18—H18	0.9800
C1—P1	1.824 (3)	C19—C20	1.515 (5)
C2—C3	1.415 (5)	C19—P2	1.850 (3)
C2—H2	0.9800	C19—H19A	0.9700
C3—C4	1.413 (5)	C19—H19B	0.9700
C3—H3	0.9800	C20—C21	1.515 (6)
C4—C5	1.421 (5)	C20—C22	1.532 (5)
C4—H4	0.9800	C20—H20	0.9800
C5—H5	0.9800	C21—H21A	0.9600
C6—C7	1.513 (5)	C21—H21B	0.9600
C6—P1	1.845 (4)	C21—H21C	0.9600
C6—H6A	0.9700	C22—H22A	0.9600
C6—H6B	0.9700	C22—H22B	0.9600
C7—C8	1.508 (6)	C22—H22C	0.9600
C7—C9	1.530 (6)	C23—C24	1.519 (5)
C7—H7	0.9800	C23—P2	1.850 (4)
C8—H8A	0.9600	C23—H23A	0.9700
C8—H8B	0.9600	C23—H23B	0.9700
C8—H8C	0.9600	C24—C25	1.487 (6)
C9—H9A	0.9600	C24—C26	1.527 (6)
C9—H9B	0.9600	C24—H24	0.9800
C9—H9C	0.9600	C25—H25A	0.9600
C10—C11	1.523 (5)	C25—H25B	0.9600
C10—P1	1.851 (4)	C25—H25C	0.9600
C10—H10A	0.9700	C26—H26A	0.9600
C10—H10B	0.9700	C26—H26B	0.9600
C11—C13	1.512 (6)	C26—H26C	0.9600
C11—C12	1.526 (5)	F1—P3	1.586 (2)
C11—H11	0.9800	F2—P3	1.585 (2)
C12—H12A	0.9600	F3—P3	1.579 (3)
C12—H12B	0.9600	F4—P3	1.584 (2)
C12—H12C	0.9600	F5—P3	1.576 (2)
C13—H13A	0.9600	F6—P3	1.585 (3)
			
C16—Co1—C2	109.48 (15)	C11—C12—H12A	109.5
C16—Co1—C18	68.21 (15)	C11—C12—H12B	109.5
C2—Co1—C18	176.26 (14)	H12A—C12—H12B	109.5
C16—Co1—C17	40.40 (14)	C11—C12—H12C	109.5
C2—Co1—C17	135.80 (16)	H12A—C12—H12C	109.5
C18—Co1—C17	40.57 (14)	H12B—C12—H12C	109.5
C16—Co1—C15	40.50 (13)	C11—C13—H13A	109.5
C2—Co1—C15	112.05 (14)	C11—C13—H13B	109.5
C18—Co1—C15	68.31 (14)	H13A—C13—H13B	109.5
C17—Co1—C15	68.08 (15)	C11—C13—H13C	109.5
C16—Co1—C5	141.94 (15)	H13A—C13—H13C	109.5
C2—Co1—C5	68.72 (14)	H13B—C13—H13C	109.5
C18—Co1—C5	111.10 (14)	C15—C14—C18	105.9 (3)
C17—Co1—C5	113.26 (15)	C15—C14—P2	130.1 (3)
C15—Co1—C5	177.38 (13)	C18—C14—P2	124.0 (3)
C16—Co1—C1	111.52 (14)	C15—C14—Co1	68.81 (18)
C2—Co1—C1	41.36 (12)	C18—C14—Co1	68.62 (18)
C18—Co1—C1	136.13 (13)	P2—C14—Co1	126.59 (17)
C17—Co1—C1	109.39 (14)	C16—C15—C14	108.8 (3)
C15—Co1—C1	141.09 (13)	C16—C15—Co1	69.44 (19)
C5—Co1—C1	41.07 (13)	C14—C15—Co1	70.28 (18)
C16—Co1—C3	136.09 (16)	C16—C15—H15	125.6
C2—Co1—C3	40.66 (14)	C14—C15—H15	125.6
C18—Co1—C3	143.01 (15)	Co1—C15—H15	125.6
C17—Co1—C3	175.89 (16)	C17—C16—C15	108.4 (3)
C15—Co1—C3	110.47 (15)	C17—C16—Co1	70.0 (2)
C5—Co1—C3	68.35 (15)	C15—C16—Co1	70.07 (19)
C1—Co1—C3	69.14 (13)	C17—C16—H16	125.8
C16—Co1—C4	176.30 (16)	C15—C16—H16	125.8
C2—Co1—C4	68.30 (14)	Co1—C16—H16	125.8
C18—Co1—C4	114.16 (15)	C16—C17—C18	108.1 (3)
C17—Co1—C4	143.21 (16)	C16—C17—Co1	69.6 (2)
C15—Co1—C4	136.99 (15)	C18—C17—Co1	69.65 (19)
C5—Co1—C4	40.65 (13)	C16—C17—H17	126.0
C1—Co1—C4	68.97 (14)	C18—C17—H17	126.0
C3—Co1—C4	40.35 (14)	Co1—C17—H17	126.0
C16—Co1—C14	68.80 (14)	C17—C18—C14	108.8 (3)
C2—Co1—C14	141.54 (13)	C17—C18—Co1	69.8 (2)
C18—Co1—C14	41.02 (12)	C14—C18—Co1	70.36 (18)
C17—Co1—C14	68.82 (14)	C17—C18—H18	125.6
C15—Co1—C14	40.91 (12)	C14—C18—H18	125.6
C5—Co1—C14	137.02 (13)	Co1—C18—H18	125.6
C1—Co1—C14	177.04 (13)	C20—C19—P2	116.2 (3)
C3—Co1—C14	112.82 (14)	C20—C19—H19A	108.2
C4—Co1—C14	110.91 (14)	P2—C19—H19A	108.2
C5—C1—C2	106.3 (3)	C20—C19—H19B	108.2
C5—C1—P1	130.4 (3)	P2—C19—H19B	108.2
C2—C1—P1	123.3 (3)	H19A—C19—H19B	107.4
C5—C1—Co1	69.38 (19)	C19—C20—C21	112.4 (4)
C2—C1—Co1	68.86 (18)	C19—C20—C22	110.6 (4)
P1—C1—Co1	124.48 (16)	C21—C20—C22	110.4 (4)
C3—C2—C1	108.7 (3)	C19—C20—H20	107.8
C3—C2—Co1	70.3 (2)	C21—C20—H20	107.8
C1—C2—Co1	69.78 (18)	C22—C20—H20	107.8
C3—C2—H2	125.6	C20—C21—H21A	109.5
C1—C2—H2	125.6	C20—C21—H21B	109.5
Co1—C2—H2	125.6	H21A—C21—H21B	109.5
C4—C3—C2	108.2 (3)	C20—C21—H21C	109.5
C4—C3—Co1	70.16 (19)	H21A—C21—H21C	109.5
C2—C3—Co1	69.06 (19)	H21B—C21—H21C	109.5
C4—C3—H3	125.9	C20—C22—H22A	109.5
C2—C3—H3	125.9	C20—C22—H22B	109.5
Co1—C3—H3	125.9	H22A—C22—H22B	109.5
C3—C4—C5	108.0 (3)	C20—C22—H22C	109.5
C3—C4—Co1	69.49 (19)	H22A—C22—H22C	109.5
C5—C4—Co1	69.10 (18)	H22B—C22—H22C	109.5
C3—C4—H4	126.0	C24—C23—P2	115.0 (3)
C5—C4—H4	126.0	C24—C23—H23A	108.5
Co1—C4—H4	126.0	P2—C23—H23A	108.5
C4—C5—C1	108.7 (3)	C24—C23—H23B	108.5
C4—C5—Co1	70.25 (19)	P2—C23—H23B	108.5
C1—C5—Co1	69.55 (18)	H23A—C23—H23B	107.5
C4—C5—H5	125.6	C25—C24—C23	113.7 (4)
C1—C5—H5	125.6	C25—C24—C26	111.2 (4)
Co1—C5—H5	125.6	C23—C24—C26	109.4 (4)
C7—C6—P1	112.9 (3)	C25—C24—H24	107.4
C7—C6—H6A	109.0	C23—C24—H24	107.4
P1—C6—H6A	109.0	C26—C24—H24	107.4
C7—C6—H6B	109.0	C24—C25—H25A	109.5
P1—C6—H6B	109.0	C24—C25—H25B	109.5
H6A—C6—H6B	107.8	H25A—C25—H25B	109.5
C8—C7—C6	111.5 (4)	C24—C25—H25C	109.5
C8—C7—C9	109.4 (4)	H25A—C25—H25C	109.5
C6—C7—C9	110.8 (4)	H25B—C25—H25C	109.5
C8—C7—H7	108.4	C24—C26—H26A	109.5
C6—C7—H7	108.4	C24—C26—H26B	109.5
C9—C7—H7	108.4	H26A—C26—H26B	109.5
C7—C8—H8A	109.5	C24—C26—H26C	109.5
C7—C8—H8B	109.5	H26A—C26—H26C	109.5
H8A—C8—H8B	109.5	H26B—C26—H26C	109.5
C7—C8—H8C	109.5	C1—P1—C6	101.32 (16)
H8A—C8—H8C	109.5	C1—P1—C10	98.64 (16)
H8B—C8—H8C	109.5	C6—P1—C10	99.83 (17)
C7—C9—H9A	109.5	C14—P2—C23	99.00 (16)
C7—C9—H9B	109.5	C14—P2—C19	98.79 (16)
H9A—C9—H9B	109.5	C23—P2—C19	99.08 (18)
C7—C9—H9C	109.5	F5—P3—F3	89.92 (16)
H9A—C9—H9C	109.5	F5—P3—F4	90.45 (15)
H9B—C9—H9C	109.5	F3—P3—F4	89.63 (15)
C11—C10—P1	115.8 (3)	F5—P3—F6	89.95 (16)
C11—C10—H10A	108.3	F3—P3—F6	179.69 (17)
P1—C10—H10A	108.3	F4—P3—F6	90.65 (16)
C11—C10—H10B	108.3	F5—P3—F2	178.99 (18)
P1—C10—H10B	108.3	F3—P3—F2	91.09 (16)
H10A—C10—H10B	107.4	F4—P3—F2	89.53 (14)
C13—C11—C10	112.2 (4)	F6—P3—F2	89.04 (16)
C13—C11—C12	110.9 (4)	F5—P3—F1	90.58 (15)
C10—C11—C12	110.1 (4)	F3—P3—F1	90.94 (15)
C13—C11—H11	107.8	F4—P3—F1	178.83 (16)
C10—C11—H11	107.8	F6—P3—F1	88.78 (14)
C12—C11—H11	107.8	F2—P3—F1	89.44 (14)
			
C16—Co1—C1—C5	−146.7 (2)	C15—Co1—C14—C18	117.8 (3)
C2—Co1—C1—C5	117.9 (3)	C5—Co1—C14—C18	−64.6 (3)
C18—Co1—C1—C5	−66.2 (3)	C3—Co1—C14—C18	−146.8 (2)
C17—Co1—C1—C5	−103.5 (2)	C4—Co1—C14—C18	−103.2 (2)
C15—Co1—C1—C5	177.7 (2)	C16—Co1—C14—P2	−162.0 (3)
C3—Co1—C1—C5	80.6 (2)	C2—Co1—C14—P2	−67.1 (3)
C4—Co1—C1—C5	37.2 (2)	C18—Co1—C14—P2	117.2 (3)
C16—Co1—C1—C2	95.4 (2)	C17—Co1—C14—P2	154.5 (3)
C18—Co1—C1—C2	175.9 (2)	C15—Co1—C14—P2	−125.0 (3)
C17—Co1—C1—C2	138.6 (2)	C5—Co1—C14—P2	52.6 (3)
C15—Co1—C1—C2	59.8 (3)	C3—Co1—C14—P2	−29.6 (3)
C5—Co1—C1—C2	−117.9 (3)	C4—Co1—C14—P2	14.0 (2)
C3—Co1—C1—C2	−37.3 (2)	C18—C14—C15—C16	0.0 (4)
C4—Co1—C1—C2	−80.6 (2)	P2—C14—C15—C16	179.5 (2)
C16—Co1—C1—P1	−21.2 (3)	Co1—C14—C15—C16	58.9 (2)
C2—Co1—C1—P1	−116.6 (3)	C18—C14—C15—Co1	−58.9 (2)
C18—Co1—C1—P1	59.3 (3)	P2—C14—C15—Co1	120.6 (3)
C17—Co1—C1—P1	22.0 (3)	C2—Co1—C15—C16	94.6 (2)
C15—Co1—C1—P1	−56.8 (3)	C18—Co1—C15—C16	−81.4 (2)
C5—Co1—C1—P1	125.5 (3)	C17—Co1—C15—C16	−37.5 (2)
C3—Co1—C1—P1	−153.9 (3)	C1—Co1—C15—C16	56.5 (3)
C4—Co1—C1—P1	162.8 (2)	C3—Co1—C15—C16	138.3 (2)
C5—C1—C2—C3	0.2 (4)	C4—Co1—C15—C16	175.9 (2)
P1—C1—C2—C3	177.9 (2)	C14—Co1—C15—C16	−120.1 (3)
Co1—C1—C2—C3	59.7 (2)	C16—Co1—C15—C14	120.1 (3)
C5—C1—C2—Co1	−59.6 (2)	C2—Co1—C15—C14	−145.4 (2)
P1—C1—C2—Co1	118.2 (2)	C18—Co1—C15—C14	38.66 (19)
C16—Co1—C2—C3	139.5 (2)	C17—Co1—C15—C14	82.5 (2)
C17—Co1—C2—C3	176.9 (2)	C1—Co1—C15—C14	176.6 (2)
C15—Co1—C2—C3	96.2 (2)	C3—Co1—C15—C14	−101.6 (2)
C5—Co1—C2—C3	−81.1 (2)	C4—Co1—C15—C14	−64.0 (3)
C1—Co1—C2—C3	−119.7 (3)	C14—C15—C16—C17	0.4 (4)
C4—Co1—C2—C3	−37.3 (2)	Co1—C15—C16—C17	59.8 (2)
C14—Co1—C2—C3	59.4 (3)	C14—C15—C16—Co1	−59.4 (2)
C16—Co1—C2—C1	−100.8 (2)	C2—Co1—C16—C17	139.3 (2)
C17—Co1—C2—C1	−63.4 (3)	C18—Co1—C16—C17	−37.6 (2)
C15—Co1—C2—C1	−144.14 (19)	C15—Co1—C16—C17	−119.3 (3)
C5—Co1—C2—C1	38.55 (19)	C5—Co1—C16—C17	59.2 (3)
C3—Co1—C2—C1	119.7 (3)	C1—Co1—C16—C17	95.0 (2)
C4—Co1—C2—C1	82.4 (2)	C3—Co1—C16—C17	176.8 (2)
C14—Co1—C2—C1	179.09 (19)	C14—Co1—C16—C17	−81.8 (2)
C1—C2—C3—C4	0.0 (4)	C2—Co1—C16—C15	−101.5 (2)
Co1—C2—C3—C4	59.4 (2)	C18—Co1—C16—C15	81.7 (2)
C1—C2—C3—Co1	−59.4 (2)	C17—Co1—C16—C15	119.3 (3)
C16—Co1—C3—C4	178.5 (2)	C5—Co1—C16—C15	178.5 (2)
C2—Co1—C3—C4	−119.6 (3)	C1—Co1—C16—C15	−145.7 (2)
C18—Co1—C3—C4	59.2 (3)	C3—Co1—C16—C15	−63.9 (3)
C15—Co1—C3—C4	140.0 (2)	C14—Co1—C16—C15	37.4 (2)
C5—Co1—C3—C4	−37.5 (2)	C15—C16—C17—C18	−0.6 (4)
C1—Co1—C3—C4	−81.7 (2)	Co1—C16—C17—C18	59.2 (2)
C14—Co1—C3—C4	95.9 (2)	C15—C16—C17—Co1	−59.8 (2)
C16—Co1—C3—C2	−61.9 (3)	C2—Co1—C17—C16	−62.0 (3)
C18—Co1—C3—C2	178.8 (2)	C18—Co1—C17—C16	119.4 (3)
C15—Co1—C3—C2	−100.4 (2)	C15—Co1—C17—C16	37.6 (2)
C5—Co1—C3—C2	82.1 (2)	C5—Co1—C17—C16	−144.8 (2)
C1—Co1—C3—C2	37.9 (2)	C1—Co1—C17—C16	−100.7 (2)
C4—Co1—C3—C2	119.6 (3)	C4—Co1—C17—C16	178.7 (2)
C14—Co1—C3—C2	−144.5 (2)	C14—Co1—C17—C16	81.8 (2)
C2—C3—C4—C5	−0.2 (4)	C16—Co1—C17—C18	−119.4 (3)
Co1—C3—C4—C5	58.5 (2)	C2—Co1—C17—C18	178.6 (2)
C2—C3—C4—Co1	−58.7 (2)	C15—Co1—C17—C18	−81.8 (2)
C2—Co1—C4—C3	37.6 (2)	C5—Co1—C17—C18	95.8 (2)
C18—Co1—C4—C3	−145.5 (2)	C1—Co1—C17—C18	139.8 (2)
C17—Co1—C4—C3	176.7 (2)	C4—Co1—C17—C18	59.3 (3)
C15—Co1—C4—C3	−62.0 (3)	C14—Co1—C17—C18	−37.7 (2)
C5—Co1—C4—C3	119.7 (3)	C16—C17—C18—C14	0.6 (4)
C1—Co1—C4—C3	82.1 (2)	Co1—C17—C18—C14	59.7 (2)
C14—Co1—C4—C3	−101.0 (2)	C16—C17—C18—Co1	−59.1 (2)
C2—Co1—C4—C5	−82.2 (2)	C15—C14—C18—C17	−0.4 (4)
C18—Co1—C4—C5	94.8 (2)	P2—C14—C18—C17	−179.9 (2)
C17—Co1—C4—C5	57.0 (3)	Co1—C14—C18—C17	−59.4 (2)
C15—Co1—C4—C5	178.3 (2)	C15—C14—C18—Co1	59.0 (2)
C1—Co1—C4—C5	−37.6 (2)	P2—C14—C18—Co1	−120.5 (2)
C3—Co1—C4—C5	−119.7 (3)	C16—Co1—C18—C17	37.4 (2)
C14—Co1—C4—C5	139.2 (2)	C15—Co1—C18—C17	81.2 (2)
C3—C4—C5—C1	0.3 (4)	C5—Co1—C18—C17	−101.6 (2)
Co1—C4—C5—C1	59.1 (2)	C1—Co1—C18—C17	−61.4 (3)
C3—C4—C5—Co1	−58.8 (2)	C3—Co1—C18—C17	176.8 (3)
C2—C1—C5—C4	−0.3 (4)	C4—Co1—C18—C17	−145.6 (2)
P1—C1—C5—C4	−177.8 (2)	C14—Co1—C18—C17	119.7 (3)
Co1—C1—C5—C4	−59.5 (2)	C16—Co1—C18—C14	−82.3 (2)
C2—C1—C5—Co1	59.2 (2)	C17—Co1—C18—C14	−119.7 (3)
P1—C1—C5—Co1	−118.3 (3)	C15—Co1—C18—C14	−38.56 (19)
C16—Co1—C5—C4	175.8 (2)	C5—Co1—C18—C14	138.70 (19)
C2—Co1—C5—C4	81.1 (2)	C1—Co1—C18—C14	178.82 (18)
C18—Co1—C5—C4	−103.0 (2)	C3—Co1—C18—C14	57.0 (3)
C17—Co1—C5—C4	−146.9 (2)	C4—Co1—C18—C14	94.6 (2)
C1—Co1—C5—C4	119.9 (3)	P2—C19—C20—C21	61.7 (5)
C3—Co1—C5—C4	37.2 (2)	P2—C19—C20—C22	−174.4 (3)
C14—Co1—C5—C4	−63.5 (3)	P2—C23—C24—C25	61.3 (5)
C16—Co1—C5—C1	56.0 (3)	P2—C23—C24—C26	−173.6 (3)
C2—Co1—C5—C1	−38.82 (19)	C5—C1—P1—C6	4.9 (3)
C18—Co1—C5—C1	137.2 (2)	C2—C1—P1—C6	−172.2 (3)
C17—Co1—C5—C1	93.2 (2)	Co1—C1—P1—C6	−86.4 (2)
C3—Co1—C5—C1	−82.7 (2)	C5—C1—P1—C10	−97.0 (3)
C4—Co1—C5—C1	−119.9 (3)	C2—C1—P1—C10	85.8 (3)
C14—Co1—C5—C1	176.62 (19)	Co1—C1—P1—C10	171.7 (2)
P1—C6—C7—C8	−67.0 (5)	C7—C6—P1—C1	171.4 (3)
P1—C6—C7—C9	171.0 (3)	C7—C6—P1—C10	−87.7 (3)
P1—C10—C11—C13	−59.7 (5)	C11—C10—P1—C1	−70.1 (3)
P1—C10—C11—C12	176.3 (3)	C11—C10—P1—C6	−173.2 (3)
C16—Co1—C14—C15	−37.1 (2)	C15—C14—P2—C23	−0.1 (3)
C2—Co1—C14—C15	57.9 (3)	C18—C14—P2—C23	179.4 (3)
C18—Co1—C14—C15	−117.8 (3)	Co1—C14—P2—C23	92.0 (2)
C17—Co1—C14—C15	−80.5 (2)	C15—C14—P2—C19	100.6 (3)
C5—Co1—C14—C15	177.6 (2)	C18—C14—P2—C19	−79.9 (3)
C3—Co1—C14—C15	95.4 (2)	Co1—C14—P2—C19	−167.2 (2)
C4—Co1—C14—C15	139.0 (2)	C24—C23—P2—C14	179.3 (3)
C16—Co1—C14—C18	80.7 (2)	C24—C23—P2—C19	78.8 (3)
C2—Co1—C14—C18	175.7 (2)	C20—C19—P2—C14	81.0 (3)
C17—Co1—C14—C18	37.3 (2)	C20—C19—P2—C23	−178.3 (3)

### Hydrogen-bond geometry (Å, °)

**Table:**

*D*—H···*A*	*D*—H	H···*A*	*D*···*A*	*D*—H···*A*
C3—H3···F5^i^	0.98	2.44	3.237 (5)	138
C15—H15···F4^i^	0.98	2.39	3.278 (4)	150
C17—H17···F2	0.98	2.51	3.188 (5)	126
C18—H18···F6	0.98	2.41	3.305 (4)	152
C19—H19B···F2^ii^	0.97	2.54	3.494 (4)	167

Symmetry codes: (i) *x*, *y*−1, *z*; (ii) −*x*+3/2, *y*−1/2, −*z*+1/2.
